# Responsive Sensory Evaluation to Develop Flexible Taste-Masked Paediatric Primaquine Tablets against Malaria for Low-Resource Settings

**DOI:** 10.3390/pharmaceutics15071879

**Published:** 2023-07-04

**Authors:** Sejal R. Ranmal, Marc Lavarde, Elodie Wallon, Samar Issa, Walter R. Taylor, Julie L. A. Nguyen Ngoc Pouplin, Catherine Tuleu, Anne-Marie Pensé-Lhéritier

**Affiliations:** 1 UCL School of Pharmacy, University College London, 29–39 Brunswick Square, London WC1N 1AX, UK; sejal.ranmal@ucl.ac.uk (S.R.R.); c.tuleu@ucl.ac.uk (C.T.); 2 Ecole de Biologie Industrielle—EBI, UPR EBInnov®, 49 Avenue des Genottes CS90009, 95895 Cergy, France; corp.wallon@hubebi.com (E.W.); s.issa@hubebi.com (S.I.); annemarielheritier@frmgalesens.fr (A.-M.P.-L.); 3 Mahidol Oxford Tropical Medicine Research Unit, Faculty of Tropical Medicine, Mahidol University, 420/60 Rajvithi Road, Bangkok 10400, Thailand; bob@tropmedres.ac; 4 Centre for Tropical Medicine and Global Health, Nuffield Department of Medicine, University of Oxford, Oxford OX1 3SY, UK; 5 Reseau Medicaments et Developpement (ReMeD), 21bis Avenue du Commandant l’Herminier, 44600 Saint-Nazaire, France; julie.nguyen@remed.org

**Keywords:** primaquine, malaria, child, paediatric formulation, palatability, acceptability, human taste panel, sensory evaluation, design of experiments, quality target product profile

## Abstract

Primaquine is an important antimalarial drug for malaria transmission blocking and radical cure, but it is not currently available in child-friendly formulations in appropriate doses. Adult-strength tablets are often crushed and dissolved in water to obtain the required dose, which exposes the drug’s bitter taste. As part of the developing paediatric primaquine (DPP) project, this study adopted a responsive sensory pharmaceutics approach by integrating real-time formulation development and pre-clinical taste assessment to develop palatable, flavour-infused primaquine tablets. A design of experiment (DoE) approach was used to screen different taste-masking agents and excipient blends with trained, expert sensory assessors, with quinine hydrochloride as a model bitter tastant. The taste-masking efficacy of selected prototype formulation blends was validated with naïve assessors using the highest 15 mg primaquine dose. The mean bitterness intensity rating, measured on a discrete 11-point scale, was halved from 7.04 for the unflavoured control to 2.74–3.70 for the formulation blends. Sucralose had the biggest impact on bitterness suppression and improving palatability. Two different flavouring systems have been developed, and their acceptability in paediatric patients will be assessed as part of upcoming validation field clinical trials in Africa.

## 1. Introduction

Primaquine is an old, low-cost generic drug that is not widely used, even though it is a vital part of the arsenal in the global fight against malaria. The World Health Organisation (WHO) estimates that nearly half of the world’s population remains at risk of this deadly yet curable disease, with young children particularly vulnerable to infection and death [1]. In 2021, the WHO African Region accounted for 95% of malaria cases and 96% of malaria deaths; around 80% of these were children under 5 years old [2]. Primaquine plays two key roles in malaria elimination: a single, low dose (SLD) is given with standard treatment to block the transmission of *Plasmodium falciparum*, while a radical cure treatment of *P. vivax* and *P. ovale* is generally given over fourteen days to kill liver hypnozoites and prevent relapses (new infections derived from hypnozoites).

An adult dose 15 mg tablet of primaquine base, as primaquine phosphate (PQ), was registered in the 1950s with The United States Food and Drug Administration (FDA), while a 7.5 mg base tablet was registered in the European Union (EU) some 10 years ago. Globally, these two tablet strengths remain the only licenced, quality-assured formulations for patients of all ages. This limited product availability prohibits appropriate age- or weight-based dosing for both clinical indications because of the inconvenience and consequent inaccuracy of using tablet fractions. There are currently no WHO-approved age- or weight-based PQ regimens, but age-dosed SLDPQ has been assessed for safety in young African children aged between six months and 11 years [3]. Five weight-based dosing regimens and four age-based regimens have been proposed for radical cure [4]. Based on the dose regimen applied, children aged six months to seven years would require lower doses; for example, a standard regimen of 0.25 mg/kg/day for a 10-kg child of 12-months age would require the 2.5 mg tablet for a single dose. Therefore, there is an urgent need to have lower-strength tablets. As such, WHO’s most recent prequalification programme Expression of Interest (EOI) list for antimalarials includes tablets of primaquine base at lower 2.5 mg and 5 mg strengths [5].

PQ is a bitter-tasting drug; therefore, in addition to suitable dosage strengths, paediatric formulations need to be palatable and acceptable to patients. Palatability is defined as “the overall appreciation of an (often oral) medicinal product in relation to its smell, taste, aftertaste, and texture (i.e., feeling in the mouth)” and is defined by the characteristics of the active substance, the formulation excipients, and how they are formulated into the finished medicinal product [6]. In practise, PQ tablets are often crushed and dissolved in water before taking the necessary volume to obtain the targeted paediatric dose; however, the product manufacturers themselves note that this is not an optimal solution since the drug is extremely bitter [7].

The aversive taste of PQ has been established across research and clinical settings. In vitro studies using artificial taste sensors with membranes sensitive to bitterness found PQ to be a basic bitter compound with taste characteristics close to quinine, one of the most well-established, model bitter-tasting substances [8,9]. In a gustatory taste sensation test, twenty healthy adult volunteers evaluated a 1 mL dispersion containing 10 mg of PQ (presumed from the methods described) by holding the sample in their mouths for 30 s, spitting it out, and rating the bitterness on an eight-point numerical scale from “0 = tasteless” to “3+ = very strong” [10]. Overall, 95% of the panel rated the drug as “3 = strongly bitter”, and 5% as “3+ = very strong” [10]. In a field study in Tanzania, concerns about the bitter taste of dissolved PQ tablets led healthcare providers to administer the medicine on a spoon mixed with a sweet beverage, mainly soda and fruit punch, to help mask the poor taste [11]. All healthcare providers reported that crushing and dissolving tablets to administer the appropriate dose was challenging, and there were concerns that the need for sweet beverages presented unnecessary additional costs to patients [11].

The Developing Paediatric Primaquine (DPP) project (www.dpp-project.org; accessed on 20 May 2023) aims to develop and deploy a palatable, child-friendly PQ formulation across a range of doses in optimised regimens for transmission blocking and radical cure in low- and middle-income countries. The main product is a full line of flavoured PQ tablets covering an expanded range of tablet strengths to answer the WHO EOI for prequalification [5]. The formulation blends will be adapted proportionally (homothetically) to develop the different tablet strengths. The African-European consortium, together with an industrial partner, plans to design, manufacture, and then validate the new paediatric formulation in clinical field studies. Table 1 outlines the initial key attributes of the intended tablet formulations based on the paediatric Quality Target Product Profile (pQTPP) proposed by an expert working group [12]. A full pQTPP for the final product, including attributes such as stability, administration protocol, packaging, and patient access, will be defined following field validation.

Sensory evaluation is a scientific method used to evaluate a product based on the five senses, and its methodologies can be used effectively to guide the development of pharmaceutical formulations. Harmonised methodologies for sensory analysis have been developed by the International Organisation for Standardisation (ISO). Sensory testing is conducted with a group of human assessors, or panellists, who evaluate specific product attributes and provide qualitative and quantitative responses. The data can be used to find effective ingredients and create optimal formulas [14]. Panels can be formed of naïve assessors who do not undertake any formal training or selection or expert assessors with demonstrated sensory sensitivity, training, and experience to make consistent and repeatable assessments [15,16]. Different sensory evaluation methods can be grouped into three main types: discriminative, descriptive, and hedonic. Discriminative tests focus on product differences and can be performed by trained or untrained assessors [17,18]. Descriptive methods aim to give global information about the product and typically require a trained expert panel to characterise specific features and score their perceived intensity. The process of training panellists takes several months to ensure that definitions, protocols, and rating scales are controlled [19]. Hedonic methods (including ranking and appreciation tests) focus on the consumer and their preferences, and rather than using expert assessors, a large sample of naïve consumers is needed to provide enough statistical power [20].

The aim of the study reported herein was to adopt a responsive sensory pharmaceutics approach to facilitate the development of a novel paediatric PQ formulation by integrating real-time formulation development and pre-clinical taste assessment. A combination of excipient blends, including sweeteners, flavouring agents, and taste modifiers, were screened as part of the taste-masking strategy, and multiple sensory evaluation panels were conducted to measure key palatability attributes. To the authors’ knowledge, this is the first time that sensory evaluation methodologies have been used from the outset to steer and optimise formulation design.

## 2. Materials and Methods

### 2.1. Materials

The main bitter-tasting active substances used in the study included PQ (IPCA Laboratories Ltd., Mumbai, India) and food-grade quinine hydrochloride (QHCl) (Sigma-Aldrich, Merck KgaA, Darmstadt, Germany). Caffeine (Horizon Nature, Villefranche Sur Saône, France), citric acid (Merck KgaA, Darmstadt, Germany), and sucrose (Daddy, Cristalco SAS, Paris, France) were also used as reference materials when training and calibrating expert sensory assessors. Bitterness sensitivity screening in naïve assessors was completed using Burghart (or ODOFIN) taste strips (MediSense, Groningen, The Netherlands).

The PQ formulation samples were based on the adult dose of 26.3 mg (15 mg primaquine base) and included the tablet core excipients (diluents, binders, and lubricants) used by the product manufacturer. Flavouring agents including strawberry (two types), orange, banana, tutti frutti, mango, vanilla, and tropical were all kindly donated by Firmenich (Firmenich SA, Geneva, Switzerland). Other excipients used in the taste-masking flavour blends included the sweeteners sucrose (Daddy, Cristalco SAS, Paris, France), maltitol (SweetPearl^®^ P300 DC Maltitol, Roquette, France), and sucralose (Tasteprint™, Firmenich SA, Geneva, Switzerland); bitter blockers including Bitter Blocker Masking™ (Firmenich SA, Geneva, Switzerland) and sodium gluconate (Jungbunzlauer, Basel, Switzerland); Vanilift™ Flavour (Firmenich SA, Geneva, Switzerland), a natural flavour ingredient to boost the vanillic taste and contribute to bitterness masking; and citric acid (Merck KGaA, Darmstadt, Germany) as a taste-modifier. Bottled water and unleavened bread used during the panel studies were purchased from local supermarkets at each site.

### 2.2. End-User Survey to Identify Flavour Preferences

An informal qualitative survey was distributed to an opportunistic, purposive sample of clinical researchers (mainly physicians) in the DPP network. These were professionals in the clinical research field in target low- and middle-income countries (LMICs) who work closely with paediatric malaria patients and would therefore provide a proxy for patient preferences. Participants were asked their opinion on flavours or aromas that they believed children would like when taking medicinal products to help guide the selection of flavouring agents for initial screening.

### 2.3. Overview of Sensory Panel Studies

Four sensory panel studies were conducted with expert assessors at the Ecole de Biologie Industrielle (EBI) in France and naïve assessors from University College London (UCL) in the UK. The design of each study is summarised in Table 2. Approvals were sought from local institutional research ethics committees. Due to differences in national regulations, panels in the UK were completed with PQ, while food-grade QHCl was used as the active bitter tastant in France. The sessions at EBI were carried out in a designated test room designed in accordance with ISO 8589 [21]. The studies at UCL were conducted in the university dispensary skills lab, a well-ventilated and odourless environment with ample daylight and free of noise and distraction to avoid any influence on sensory evaluation.

### 2.4. Training and Selection of Sensory Assessors

All sensory assessors were healthy male and female young adults with a mean age between 21 and 24 years (range 18–34 years) across all panel studies. Volunteers were provided with detailed participant information sheets, and informed consent forms were signed by all assessors. Participants were excluded if they had dysgeusia or any sensory disorders affecting taste and smell, any drug allergies, any symptoms of COVID-19, or had any dental care or medicinal treatment (excluding contraceptives and over-the-counter medicines) up to 15 days before the study.

#### 2.4.1. Expert Assessors, EBI

Volunteers, who were second-year students at EBI, were selected and trained as assessors using methodologies described in ISO standards. Initially, bitterness sensitivity screening was completed using local procedures based on the ISO 8587 ranking test [22]. Participants evaluated solutions containing 0 g/L, 0.54 g/L, and 1.8 g/L of caffeine, and those who ranked the samples in the correct order were invited to participate in the panel studies. These participants completed several training sessions in accordance with ISO 8586 on the selection, training, and monitoring of expert sensory assessors [15]. This included the qualification and quantification of specific taste attributes on a discrete 11-point scale from 0 to 10. Reference solutions included 0.108 g/L QHCl for bitterness (with a designated value of 6 on the scale), 0.720 g/L of citric acid for sourness, and 12 g/L of sucrose for sweetness (both with a value of 4 on the scale). After monitoring their performance to ensure assessments were discriminatory and reproducible, participants were qualified as expert assessors. As this study took place over two successive academic years, the recruitment, training, and qualification of experts were carried out twice.

#### 2.4.2. Naïve Assessors, UCL

Volunteers, who were university students, completed a simple screening to determine their bitterness sensitivity using one blank strip (containing no tastant) and one bitter strip (impregnated with 6 mg/mL QHCl). Participants evaluated each strip by placing it on the front middle region of the tongue for 10 s. Each strip was blinded and randomised, and water was consumed before and after each evaluation. Those with the correct rank order were invited to take part in the panel studies.

### 2.5. Sample Evaluation

In all panel studies, 10 mL liquid samples were evaluated using the “swirl and spit” technique. Participants swirled the sample around the mouth, covering all oral surfaces (under the tongue and against the cheeks) for 10 s before spitting it out into a receptacle. Samples were blinded using a 3- or 4-digit product code and presented in a randomised order generated according to the Latin-square Williams Design Plan [23].

After spitting out the sample, participants rated its sensory attributes (perceptible characteristics) with intensity values to establish a sensory profile, as outlined in ISO 13299 [24]. Table 2 outlines the attributes rated in each sensory panel, including bitterness, sweetness, and sourness. An aftertaste was reported 1 min after spitting out the sample. Responses were recorded on an 11-point numerical, unipolar, discrete scale from, for example, 0 (“Not Bitter/Sweet/Sour”) to 10 (“Bitter/Sweet/Sour”). In panels II and IV, participants were asked to rate how acceptable they would find the sample as a medicine using a 5-point facial hedonic scale. The first graphic on the scale expressed a neutral expression, while each subsequent expression became more negative.

Between sample evaluations, participants were instructed to rinse and neutralise their mouths with water at least three times and consume plain crackers to remove any residual taste sensations. The researcher instructed participants to complete a neutralisation after each minute for three minutes to achieve the minimum level of neutralisation; however, participants were able to rinse and drink water as much as required. An inter-presentation interval of at least 5 min was observed between samples.

### 2.6. Statistical Analysis

Two types of statistical methodologies were used in this study: one related to the research methodology for experimental designs and the other for the statistical processing of data from the sensory panels.

Design of experiment (DoE), a set of methods for validating a hypothesis using statistical models, was used in Panel I. A screening plan was carried out to investigate the role of the taste-masking excipients, comparing their individual roles and the synergy between the factors of variation—in this study, the quantities of the raw materials. The DoE was a complete two-level factorial design with central repetition, according to Montgomery (2001) [25]. Table 3 shows the experiment matrix (as encoded by Box) and the three experimental factors: Vanilift™, sucralose, and citric acid (expressed in %). For the purposes of this study, the minimum and maximum concentrations of these excipients were chosen as follows: 1.1% ≤ Vanilift™ ≤ 2.2%; 0.5% ≤ sucralose ≤ 1.0%, and 2.0% ≤ citric acid ≤ 4.0%. The realised design was based on a two-level full factorial (2^3^) design with a central point to test the repeatability and curvature of the effects model.

Design-Expert^®^ software (version 12; Stat-Ease, Inc., Minneapolis, MN, USA) was used to generate and compute the models and statistics. In addition to the analysis of Variance (ANOVA), a Pareto chart of the effects was drawn. This statistical treatment is specific to DoEs based on Hadamard matrices and consists of using studentised effects to compare the main effects and interactions. This made it possible to classify the effects and compare them to zero using a student quantile and to compare two effects using the Bonferroni threshold.

**Figure 1:**
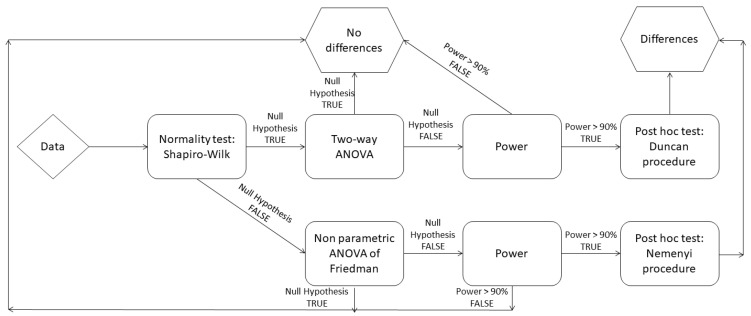
Validation process for the statistical methods.

Statistical analysis of the results of sensory tests involved applying an overall model to identify possible differences between samples (Figure 1). The statistical processing was carried out using XLStat^®^ software (version Sensory 2022.2.1, Addinsoft, NY, USA) except for the power study, which was carried out by R (version 4.1.1) and the pwr package (version 1.3-0), adapting the methods developed by Cohen (2013) [26].

The choice of the initial model depended on the assumption of normality using the Shapiro-Wilk test. If the null hypothesis was invalidated, a nonparametric approach such as Friedman’s ANOVA was used, whereas if the null hypothesis was validated, then ANOVA was used. By design, when the null hypothesis was decided, the risk of error was controlled. However, when the null hypothesis was rejected, the associated risk of error had to be assessed by means of a power study. When the models showed differences, post hoc tests were used, including the Duncan procedure when normality was validated or the Nemenyi procedure with nonparametric tests. The number of assessors for each panel study was evaluated using power studies. Sampling tables were produced to define the minimum sample size according to the power and number of formulation samples tested.

## 3. Results

### 3.1. End-User Survey

**Figure 2:**
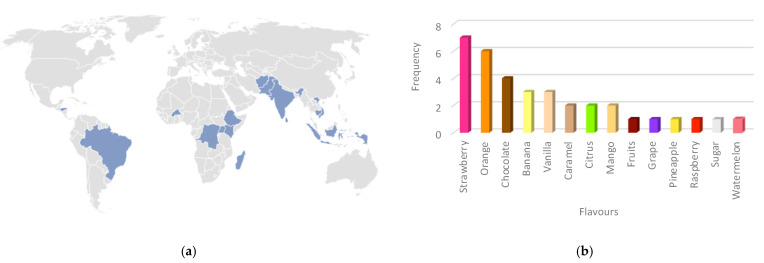
Results of the end-user survey, including (**a**) countries where survey respondents were located and (**b**) frequency of flavours reported by respondents.

The qualitative survey was completed by 20 clinicians and researchers from 14 countries in Africa, Asia, and South America (Figure 2). Fourteen different flavours were suggested, most frequently strawberry (*n =* 7), orange (*n =* 6), and chocolate (*n =* 4).

Eight flavouring agents were selected for the initial screening panel in accordance with the results of the survey: strawberry, orange, banana, tutti frutti, mango, vanilla, and tropical. Although it was not reported in the survey, tropical was a recommended flavour available from Firmenich, which also complemented the orange colour of PQ. Chocolate was not selected as it was considered an uncommon, luxury item in the target low-resource settings and therefore a flavour patients may be unfamiliar with.

### 3.2. Panel I: DoE for Screening Flavours

The screening plan was set up in three stages: two preliminary tests were completed to validate the choice of factors (selection of the optimal flavouring agents, sweeteners, and excipients) before conducting the screening plan itself.

Initially, the expert assessors evaluated samples containing 0.108 g/L of QHCl and the individual flavouring agents at increasing percentages of 1 to 3% of the total tablet weight. When tested alone, the flavouring agents showed a marginal decrease in bitterness intensity that quickly plateaued, indicating low taste-masking efficacy. There were no significant differences between the different flavouring agents. The assessors also evaluated the taste of the tablet core excipients with and without the inclusion of QHCl. The tablet core excipients had no influence on bitterness, nor did they exhibit any sweet taste sensations in themselves.

In the second step, flavour blends containing additional excipients, including sweeteners and bitter blockers, were evaluated. The bitter blockers used were not efficacious in this study. A small but significant 1-point decrease in bitterness intensity was observed when the flavouring agents were used in combination with other excipients. Samples with sweetening agents showed a more efficient dose-dependent response; however, the quantities of sucrose required were incompatible with the production of a paediatric tablet; therefore, sucralose was selected. At a quantity of 1% of the tablet weight, sucralose produced a 2-point decrease in bitterness. Ratings also improved for samples combining the flavouring agents with citric acid and Vanilift™.

**Figure 3:**
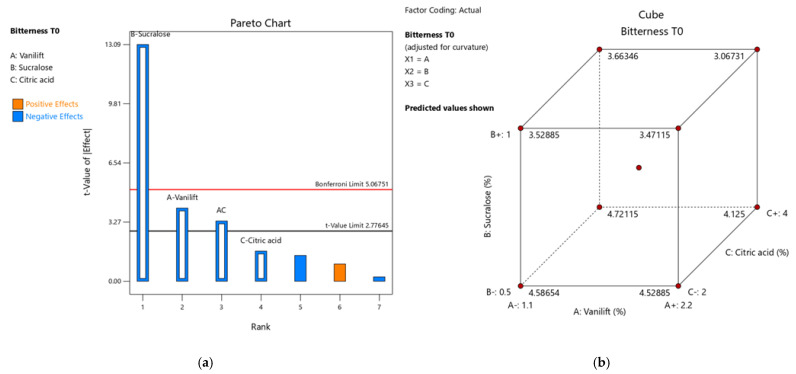
(**a**) Pareto chart of bitterness for the main effect model; and (**b**) Cube plot with mean of bitterness for the trials of the design.

The strategy then evolved into developing a more complex blend of several flavouring agents, sweeteners, and citric acid. Two leading flavouring agents were evaluated: tropical and strawberry plus vanilla. Nine combinations were prepared for the DoE with a fixed concentration of flavouring agents at 3%, sucralose at 0.5–1%, Vanilift™ at 1.1–2.2%, and citric acid at 2–4%. As shown in Figure 3, a Pareto analysis of the results showed that sucralose remained the most influential excipient in reducing bitterness, followed by Vanilift™. The lowest bitterness value (3) was observed with the highest concentrations of sucralose, Vanilift™, and citric acid, the combination resulting in a synergistic effect on taste-masking activity compared to the flavouring agents alone.

### 3.3. Panel II: Verification of Preliminary Flavour Blends

**Figure 4:**
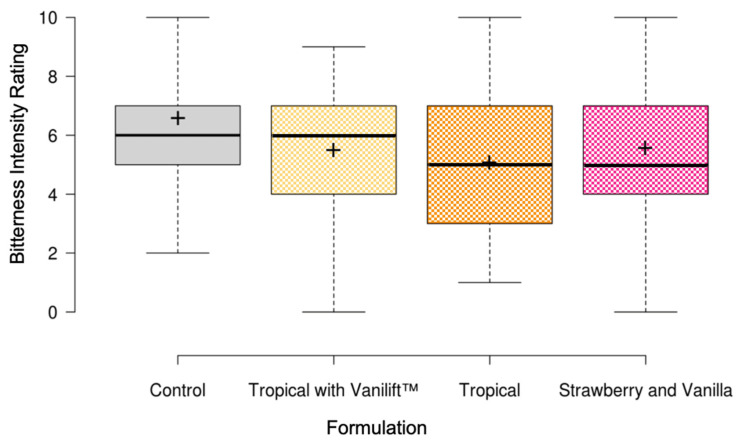
Boxplots illustrating the bitterness intensity ratings for the flavour blend formulations. Centre lines show the medians, and crosses represent the sample means. Box limits indicate the 25th and 75th percentiles, and whiskers extend 1.5 times the interquartile range.

The naïve sensory assessors evaluated a control sample of 2.63 mg/mL PQ (equivalent to 15 mg primaquine base in 10 mL) and three flavour blends based on the outcomes of the DoE. This included tropical flavour with Vanilift™, tropical flavour only, and the combination of strawberry and vanilla. The flavour blend samples also contained the tablet core excipients, sucralose, and citric acid. As shown in Figure 4, the flavour blend formulations had mean bitterness intensity ratings between 5.1 and 5.6, which were not significantly different from the 6.6 mean rating for the control. Similarly, there were no significant differences in the bitter aftertaste ratings or acceptability scores between all samples and the control.

### 3.4. Panel III: Optimisation of Flavour Blends

To optimise the flavour blends with expert panellists, the quantity of QHCl in the samples was increased to 0.132 g/L, which elicited a mean bitterness value of 8.3. This was higher than observed for the 0.108 g/L QHCl used in panel I and closer to the ratings for the PQ control sample evaluated in panel II. As a second step, increased amounts of sucralose at 2% and 3.5% were evaluated alone with QHCl. The mean bitterness ratings were reduced to 5.4 and 4.2, respectively, while sweetness ratings were similar at 8.2 and 8.7. Increased amounts of sucralose were then evaluated with the three flavour blends, which included citric acid and Vanilift™ at the same levels used in the previous two panels. When 3.5% sucralose was included in the blends, the mean bitterness ratings were reduced further to around half of the reference QHCl solution. No significant difference was found between the three blends in terms of bitterness ratings (3.4–4.0), sweetness ratings (7.2–7.4), and sourness ratings (1.1–1.9) at an α risk of 5%.

Finally, four flavour blend samples with increased quantities of sucralose up to 10% were evaluated. Bitterness ratings decreased marginally (2.9–3.2). Overall, there were no significant differences in the perception of bitterness or sweetness between the four samples. Post-hoc tests showed that sourness increased with increasing levels of sucralose; however, all intensity scores remained below 2. Based on these results, the flavour blend composition was optimised for a higher quantity of sucralose (below 10%).

### 3.5. Panel IV: Validation

**Figure 5:**
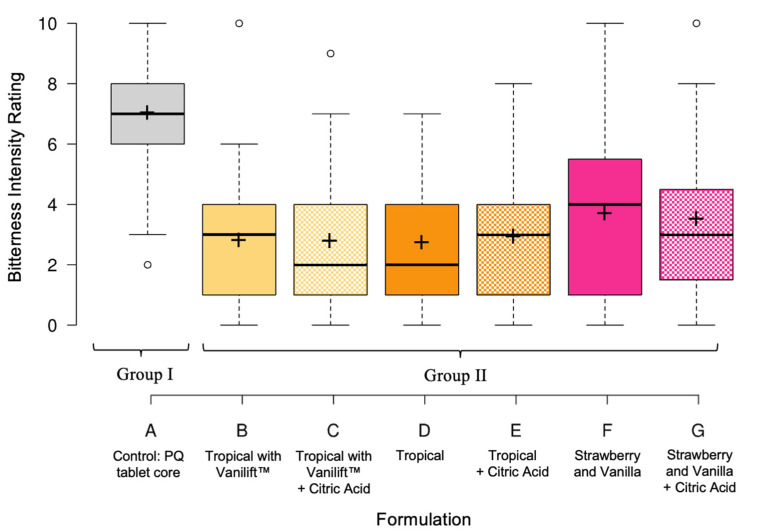
Boxplots illustrating the bitterness intensity ratings for the optimised formulations. Nemenyi’s post-hoc test classified formulation A into group I and formulations B–G into group II, indicating that all flavour blends were significantly less bitter than the control; however, there were no significant differences between the six different flavour blends. Centre lines show the medians; crosses represent the sample means; and outliers are represented by dots. Box limits indicate the 25th and 75th percentiles, and whiskers extend 1.5 times the interquartile range.

In the final validation panel with naïve assessors, the influence of citric acid was evaluated by including formulation samples with and without this taste modifier. As shown in Table 4 and Figure 5, the mean bitterness ratings of the six flavour blend formulations were approximately half of the PQ control, demonstrating bitterness taste-masking efficiency. Post-hoc multiple pairwise comparisons using Nemenyi’s procedure classified the formulations into two groups, indicating that the control formulation A was perceived as more bitter than the flavour blends; however, there was no statistically significant difference between the six different flavour blend formulations themselves.

For aftertaste, the control sample was significantly more bitter than flavour blend formulations B to F, but not G. There was no statistically significant difference in aftertaste between formulations B and F. Similarly, there was no statistically significant difference in the sweetness between the six flavour blends, although all were expectedly sweeter than the control. There was no difference in the perceived sourness between all seven formulations, including the control.

**Figure 6:**
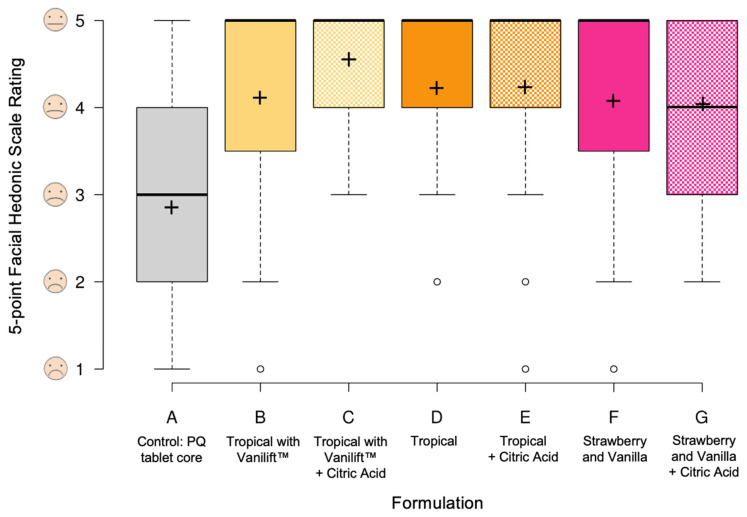
Boxplots illustrating the ratings for the overall sensory experience/sample acceptability captured on the 5-point facial hedonic scale. Centre lines show the medians; crosses represent the sample means; and outliers are represented by dots. Box limits indicate the 25th and 75th percentiles, and whiskers extend 1.5 times the interquartile range.

Figure 6 shows the results of the overall sensory experience, captured on the 5-point facial hedonic scale, to the question "How acceptable would you find the sample if it were a medicine?”. Flavour blends B to F were all rated as more acceptable than control sample A, while there was no significant difference between the control sample and formulation G. For blends B to F, over 51.9% of all assessors selected the neutral expression.

The addition of citric acid in formulations C, E, and G did not demonstrate any palatability benefits compared to the corresponding blends without this excipient. Similarly, the addition of Vanilift™ did not demonstrate any palatability benefits compared to the sample with the tropical flavouring agent alone. Collectively, the results of this study showed that the use of sucralose as a sweetener and the flavouring agents tropical (formulation D) and strawberry and vanilla (formulation F) were the optimal blends for the development of the PQ-flavoured tablet.

## 4. Discussion

In this study, we have shown how sensory evaluation techniques were instrumental in guiding the development of palatable flavouring blends of PQ, with the aim of manufacturing tablets in a range of suitable dosage strengths to treat and prevent malaria in children. Our strategy started at the bedside by first understanding the experiences and preferences of stakeholders in the field, then moved to the benchside, where a DoE approach was used to screen different taste-masking agents and excipient blends with expert sensory assessors. We validated the improved palatability of the optimised prototypes with naïve (untrained) assessors. The two leading formulation blends will be manufactured by the DPP consortium’s industry partner as tropical and strawberry/vanilla-flavoured tablets. The acceptability of these new products will be evaluated in paediatric patients back at the bedside (in comparison to the existing, simple, unflavoured tablet product) using the ClinSearch Acceptability Score Test (CAST) [13] as part of upcoming validation field trials in Ethiopia and Burkina Faso.

This approach is in line with guidance from the European Medicines Agency (EMA), which highlights that early palatability data can be acquired from dedicated adult panels, although patient acceptability of the final medicinal product should ideally be studied in end-users as an integral part of clinical studies [6]. Even for medicines designed for children, the use of dedicated sensory panels with adult assessors remains the current “gold standard” approach [27]. Recently, adult panels have been used to evaluate marketed paediatric products [28] and as part of pharmaceutical development [29,30,31] and Phase I studies [32] of medicines designed for children. In the present case, palatability was prioritised from the inception of the DPP project, and the assessment and optimisation of organoleptic properties wholly steered the design of the tablet formulation.

Sensory studies were completed with the highest adult strength (15 mg primaquine base), as this is the dose that will invoke the largest degree of bitterness when the tablet is crushed (i.e., the worst-case scenario). The samples tested in the final panel included all tablet core excipients and the flavour blends as a suspension of 2.63 mg/mL PQ (equivalent to 15 mg primaquine base in 10 mL). The anticipated lower-strength tablets (2.5 mg, 3.75 mg, 5 mg, and 7.5 mg primaquine) will be homothetic preparations from the 15 mg dry mix.

A range of taste-masking approaches exist to help overcome the aversive taste of active pharmaceutical ingredients (APIs), and these have been well reviewed elsewhere [33,34]. Many techniques can be broadly classified into those aiming to block taste transmission pathways or by preventing the release of the drug in the oral cavity (e.g., by creating a molecular or physical barrier around the API or dosage form).

Shah and Mashru (2008) describe the development of an oral reconstitutable powder of PQ through a physical mixture with cyclodextrins (CDs) [10]. CDs are naturally occurring compounds that encapsulate APIs, thus impeding their interaction with the taste buds. Evaluation by human panellists demonstrated the dry suspension powder provided taste masking; however, the dose was equivalent to 7.5 mg primaquine base in 10 mL of water (half the dose tested in the present study) [10]. The same authors also describe the development of rapidly disintegrating tablets (RDT) of PQ through solid dispersion with a salt of glycyrrhizin as a hydrophilic carrier and croscarmellose sodium as a superdisintegrant in the directly compressed tablet [35]. The solid dispersion and RDT prototypes masked the taste of the drug, again at the 7.5 mg primaquine base dose [35].

Using a judicious choice of excipients, including flavouring agents and sweeteners, is a conventional and relatively cost-effective taste-masking strategy. The simplicity, economical benefits, and practicality from a manufacturing perspective were important considerations in this project. PQ is an old and inexpensive drug; therefore, it was imperative that the final reformulated tablet would remain affordable and accessible for patients in low-resource settings.

Sucralose had the biggest impact on bitterness suppression and improving palatability. It is an artificial disaccharide and high-intensity, non-nutritive sweetener that is on average 600 times sweeter than sucrose [36]. In addition to its favourable organoleptic and sensory qualities, the physico-chemical properties of sucralose also lend themselves well to pharmaceutical applications. Sweeteners are highly water-soluble and readily dissolve in saliva to interact with taste receptors, which consequently send signals to brain centres involved in taste perception. This helps to obscure the aversive taste of the API. A strong body of evidence shows that children have higher sweet taste detection thresholds and prefer higher intensities of sweetness compared to adults [37,38,39,40]. This innate biological mechanism enables babies to detect their mother’s milk and can be linked to the growing child’s need for calories [38]. Indeed, regulators also acknowledge that sweetness often plays an important role in ensuring adequate patient acceptability of oral preparations [6].

Flavouring agents (flavours and aromatic substances) inherently have a pleasant taste and odour that can help shift patients attention away from bitterness. Two different flavouring systems have been developed herein for testing in the field, providing the opportunity for patient preferences to be considered when selecting the final product. Physiologically acceptable acids, such as citric acid, can be used with sucralose to increase its taste masking efficiency [41]; however, no significant differences in bitterness suppression or added sweetness were observed when this excipient was included in the flavouring blend. Similarly, the final sensory panel demonstrated that Vanilift™ did not demonstrate any additional benefits to palatability. Although the DoE identified a synergistic effect of bitterness reduction with the combination of Vanilift™ and citric acid, this was not observed in the final validation panel; however, the influential impact of sucralose was confirmed. The exclusion of citric acid and Vanilift™ from the final blends will have benefits in reducing costs as well as decreasing the size and weight of the tablets.

Nevertheless, although our chosen taste-masking approach can be effective, it has potential limitations. The use of sweeteners and flavouring agents in formulations reduces but seldom eliminates the bitter or aversive taste of APIs. It is difficult to predict which combinations and levels of excipients may work, and optimisation is needed on a case-by-case basis [33]. In this study, QHCl was used as a model bitter agent in the screening and optimisation studies for two principal reasons: First, quinine is a food-grade compound that is permitted for direct addition to food for human consumption, typically as a bittering agent in tonic-type drinks. As the first panel involved screening a large number of prototype formulations with the expert assessors, it was advantageous from a safety and ethical perspective to use this compound to avoid repeatedly exposing participants to the API. Second, differences in national regulations in the UK and France also needed to be considered. The sensory studies in France could not be conducted with the API itself, whereas in the UK, “swirl and spit” studies fall outside the remit of a clinical trial and could be conducted with PQ in the academic institution following local ethical approval. In light of this, the study was designed so that the formulation development benefited from the expertise and experience of the assessors in France, whose acuity enabled them to make reliable comparative judgements between initial prototypes, as well as the naïve assessors in the UK, whose untrained hedonic responses were considered more representative of the end-user. Nevertheless, the taste profiles of PQ and QHCl are not identical; therefore, it was not possible to fully extrapolate taste-masking efficiency. Benchmarking the unknown API taste (PQ) with the known API (QHCl) from the outset could have made the formulation optimisation process leaner.

Although the bitter taste intensity of PQ was significantly reduced with the flavouring blends, the continued presence of this taste sensation may impact formulation acceptability in children with malaria. Indeed, like sweetness, bitterness sensitivity is also known to vary between children and adults [38]. Flavour preferences can also vary from country to country and be influenced by patient demographics and socio-cultural background [33]. While this variability is inherent to the individual sensory experience and acceptability is a multi-faceted concept, using CAST as part of the field validation studies will help capture the real-life appreciation of the new formulation.

Before the DPP project started, PQ manufacturers and members of malaria programmes agreed that a child-friendly, taste-masked, lower-strength formulation was needed, but also that the development of new formulations is expensive and time-consuming [7]. Many essential, life-saving medicines can be optimised to improve their acceptability and clinical use; however, without funding and incentives to address these urgent challenges, vulnerable patients remain neglected. Patient and end-user acceptability of any medicine is essential and can determine whether a medical treatment is going to be effective. Non-acceptance of a medicine due to bad taste can have detrimental consequences, particularly for life-threatening conditions such as malaria, where poor adherence could lead to the development of severe malaria and possible death. Studies with antimalarial drugs have demonstrated that bitter taste can be a significant barrier to administration and, consequently, medication adherence [42,43,44]. Moreover, caregivers have even reported needing to force-feed, bribe, restrain, and even threaten children to administer poor-tasting antimalarial drugs [44]. With increasing calls to eliminate malaria around the world, there is now, more than ever, an urgent need for a PQ formulation designed for children to help fight this preventable, treatable disease.

## Figures and Tables

**Table 1:** Key formulation attributes of a paediatric Quality Target Product Profile (pQTPP) for PQ.

Attribute	Targets	Comments
Target population (age)	Entire range 6 months–<16 years	Different dosing regimens are used, depending on the endemic area and national recommendations, based on mg per body weight or by age or weight dosing bands.
Route of administration	Oral	As per the WHO EOI requirement for prequalification [5].
Target release profile	Immediate release	As per the WHO EOI requirement for prequalification [5].
Dosage form	Tablets	At the start of DPP, the 17th WHO EOI invitation in 2019 called for PQ “tablets” only, while the latest 2023 EOI adds “(preferably dispersible for paediatric use)”. Hard tablets are suitable for use in the target population; however, the tablets would need to be scored and/or crushed to be administered to children unable to swallow them whole.
Dose and dose flexibility	Paediatric dosage strengths of primaquine base: 2.5 mg 3.75 mg 5 mg (scored) 7.5 mg (scored) 15 mg (scored)	At the start of DPP, the 17th WHO EOI invitation called for prequalification of five tablet strengths; however, the latest EOI no longer lists the 3.75 mg tablet. Five strengths will continue to be developed, providing more flexibility for clinical supplies. Scorelines allow further dosing flexibility. Dose regimens require further investigation and optimisation. Clinical trials are ongoing or planned. Dosing range recommendations are developed according to the patient’s age or weight. For fixed dose combinations, if any, the ratio of active ingredients may change across age groups.
Excipients and manufacturing	Excipients: no safety concerns for the proposed patient population. All dosage strengths are to be homothetic. Low-cost manufacturing	All excipients are Generally Recognised as Safe (GRAS) and have approval for use in pharma applications in accordance with EU and African regulations. The safety of excipients for the selected age group has been considered on a risk-benefit basis, on top of regulatory acceptance and precedence. Lower strengths will be produced proportionally to the higher strengths to get a biowaiver for prequalification (i.e., a bioequivalence study is not required for the line extension). Tablets are simple to manufacture. All prices are taken in consideration to keep the finished product low-cost.
Patient acceptability	Acceptable for the proposed patient population/caregiver, and disease state.	The flavoured tablets aim to mask the bitter taste of PQ, a key strategy to improve acceptability. Sensory evaluation has been used to guide formulation development and determine palatability. Acceptability studies of the flavoured tablets versus the basic formulation will be nested in two validation field trials conducted in Ethiopia and Burkina Faso using the ClinSearch Acceptability Score Test (CAST) [13].
Administration considerations	Ease of preparation and accurate administration with low risk of dosing errors, a minimum of manipulations (e.g., mixing with water), and no device requirements.	Easy crushing is a target criterion with the aim of using a minimum of water and avoid mixing with other vehicles. The Administration protocol will be confirmed in the following field trials. No specific dosing device is needed for the dose ranges, as the various strengths ensure ease and accuracy of dosing. This mitigates the cost of a device.

**Table 2:** Overview of four sensory panel studies conducted with expert and naïve assessors to design the flavouring PQ tablet formulation.

	Panel I: Screening	Panel II: Verification	Panel III: Optimisation	Panel IV: Validation
Aim	Two-level factorial Design of Experiment (DoE) to screen combinations of flavouring agents, sweeteners, and excipient blends for bitter taste masking	Evaluation of the three leading flavour blends resulting from the DoE	Optimisation of the flavour blends and sensory profiling to quantify bitterness and other sensory attributes	Evaluation of the optimised flavoured blends, including hedonic to select two to pursue for clinical field studies.
Active Substance	QHCl	PQ	QHCl	PQ
Assessors	Expert, *n* = 26	Naïve, *n* = 17	Expert, *n* = 27	Naïve, *n* = 27
Selection and Recruitment	Formal selection and training as per ISO 8586 [15]	Simple bitterness sensitivity screening using taste strips	Formal selection and training as per ISO 8586 [15]	Simple bitterness sensitivity screening using taste strips.
Sample Assessment	Swirl and spit 10 mL of liquid samples for 10 s.			
Outcome Measures	Intensity of bitter, sweet, and sour taste attributes rated on an 11-point scale.	Intensity of bitter taste and aftertaste rated on an 11-point scale. Acceptability rated on a 5-point facial hedonic scale.	Intensity of bitter, sweet, and sour taste attributes rated on an 11-point scale.	Intensity of bitter, sweet, and sour taste and bitter aftertaste rated on an 11-point scale. Acceptability rated on a 5-point facial hedonic scale.
Setting	Sensory analysis test rooms, EBI, France	Dispensary skills lab UCL, UK	Sensory analysis test rooms, EBI, France	Dispensary skills lab UCL, UK

**Table 3:** Design of experiment (DoE) of the nine flavoured prototype formulations.

	Factor 1	Factor 2	Factor 3	Factor Levels		
**Std**	A: Vanilift^TM^	B: Sucralose	C: Citric acid	Factor (units %)	Low (−1)	High (+1)
**1**	−1	−1	−1	A: Vanilift^TM^	1.1	2.2
**2**	1	−1	−1	B: Sucralose	0.5	1.0
**3**	−1	1	−1	C: Citric acid	2.0	4.0
**4**	1	1	−1			
**5**	−1	−1	1			
**6**	1	−1	1			
**7**	−1	1	1			
**8**	1	1	1			
**9**	0	0	0			

**Table 4:** Mean taste attribute rating scores for the optimised PQ flavour blend formulations.

Formulation	Bitterness Mean	Sweetness Mean	Sourness Mean	Bitter aftertaste Mean
A: Control: PQ tablet core	7.04	0.56	2.56	3.74
B: Tropical with Vanilift™	2.81	5.37	1.19	1.44
C: Tropical with Vanilift™ + CA ^1^	2.78	5.15	2.00	1.07
D: Tropical	2.74	5.26	1.74	1.04
E: Tropical + CA ^1^	2.93	5.15	2.22	1.56
F: Strawberry and Vanilla	3.70	5.30	1.81	1.15
G: Strawberry and Vanilla + CA ^1^	3.52	5.33	2.41	1.63

^1^ CA: Citric acid.

## Data Availability

Data available on request due to confidentiality restrictions in tablet formulation.

## References

[1] (2023). WHO Guidelines for Malaria.

[2] World Health Organization (2022). World Malaria Report 2022.

[3] Taylor W.R., Olupot-Olupot P., Onyamboko M.A., Peerawaranun P., Weere W., Namayanja C., Onyas P., Titin H., Baseke J., Muhindo R. (2023). Safety of age-dosed, single low-dose primaquine in children with glucose-6-phosphate dehydrogenase deficiency who are infected with Plasmodium falciparum in Uganda and the Democratic Republic of the Congo: A randomised, double-blind, placebo-controlled, non-inferiority trial. Lancet Infect. Dis..

[4] Taylor W.R., Hoglund R.M., Peerawaranun P., Nguyen T.N., Hien T.T., Tarantola A., von Seidlein L., Tripura R., Peto T.J., Dondorp A.M. (2021). Development of weight and age-based dosing of daily primaquine for radical cure of vivax malaria. Malar. J..

[5] World Health Organization 21st Invitation to Manufacturers of Antimalarial Medicines to Submit an Expression of Interest (EOI) for Product Evaluation to the WHO Prequalification Unit (PQT). https://extranet.who.int/pqweb/sites/default/files/documents/EOI-MalariaV21_0.pdf.

[6] (2013). Guideline on Pharmaceutical Development of Medicines for Paediatric Use.

[7] Chen I., Poirot E., Newman M., Kandula D., Shah R., Hwang J., Cohen J.M., Gosling R., Rooney L. (2015). An assessment of the supply, programmatic use, and regulatory issues of single low-dose primaquine as a Plasmodium falciparum gametocytocide for sub-Saharan Africa. Malar. J..

[8] Haraguchi T., Okuno T., Nishikawa H., Kojima H., Ikegami S., Yoshida M., Habara M., Ikezaki H., Uchida T. (2019). The Relationship between Bitter Taste Sensor Response and Physicochemical Properties of 47 Pediatric Medicines and Their Biopharmaceutics Classification. Chem. Pharm. Bull..

[9] Kojima H., Kurihara T., Yoshida M., Haraguchi T., Nishikawa H., Ikegami S., Okuno T., Yamashita T., Nishikawa J., Tsujino H. (2021). A New Bitterness Evaluation Index Obtained Using the Taste Sensor for 48 Active Pharmaceutical Ingredients of Pediatric Medicines. Chem. Pharm. Bull..

[10] Shah P.P., Mashru R.C. (2008). Formulation and evaluation of taste masked oral reconstitutable suspension of primaquine phosphate. AAPS PharmSciTech.

[11] Mosha D., Kakolwa M.A., Mahende M.K., Masanja H., Abdulla S., Drakeley C., Gosling R., Wamoyi J. (2021). Safety monitoring experience of single-low dose primaquine co-administered with artemether-lumefantrine among providers and patients in routine healthcare practice: A qualitative study in Eastern Tanzania. Malar. J..

[12] Walsh J., Schaufelberger D., Iurian S., Klein S., Batchelor H., Turner R., Gizurarson S., Boltri L., Alessandrini E., Tuleu C. (2022). Path towards efficient paediatric formulation development based on partnering with clinical pharmacologists and clinicians, a conect4children expert group white paper. Br. J. Clin. Pharmacol..

[13] Ruiz F., Vallet T., Pensé-Lhéritier A.M., Aoussat A. (2017). Standardized method to assess medicines’ acceptability: Focus on paediatric population. J. Pharm. Pharmacol..

[14] Pensé-Lhéritier A.M. (2015). Recent developments in the sensorial assessment of cosmetic products: A review. Int. J. Cosmet. Sci..

[15] (2012). Sensory Analysis—General Guidelines for the Selection, Training and Monitoring of Selected Assessors and Expert Sensory Assessors.

[16] (2016). Sensory Analysis—Vocabulary—Amendment 1.

[17] Lawless H.T., Heymann H. (2010). Discrimination Testing. Sensory Evaluation of Food: Principles and Practices.

[18] Ares G., Varela P. (2017). Trained vs. consumer panels for analytical testing: Fueling a long lasting debate in the field. Food Qual. Prefer..

[19] Murray J.M., Delahunty C.M., Baxter I.A. (2001). Descriptive sensory analysis: Past, present and future. Food Res. Int..

[20] Mammasse N., Schlich P. (2014). Adequate number of consumers in a liking test. Insights from resampling in seven studies. Food Qual. Prefer..

[21] (2014). Sensory analysis—General guidance for the design of test rooms—Amendment 1.

[22] (2013). Sensory Analysis—Methodology—Ranking—Amendment 1.

[23] Jones R. (2002). Design and Analysis of Experiments (fifth edition), Douglas Montgomery, John Wiley and Sons, 2001, 684 pages, £33.95. Qual. Reliab. Eng. Int..

[24] (2016). Sensory Analysis—Methodology—General Guidance for Establishing a Sensory Profile.

[25] Montgomery D.C. (2001). Design and Analysis of Experiments.

[26] Cohen J. (2013). Statistical Power Analysis for the Behavioral Sciences.

[27] Clapham D., Bennett J., Cram A., Discihnger A., Inghelbrecht S., Pense-Lheriter A.M., Ruiz F., Salunke S., Schiele J., Soto J. (2021). Proposed Tool to Compare and Assess the Applicability of Taste Assessment Techniques for Pharmaceuticals. J. Pharm. Sci..

[28] Mennella J.A., Mathew P.S., Lowenthal E.D. (2017). Use of Adult Sensory Panel to Study Individual Differences in the Palatability of a Pediatric HIV Treatment Drug. Clin. Ther..

[29] Orubu S., Kendall R.A., Sheng Y., Tuleu C. (2022). Evaluating the Taste Masking Ability of Two Novel Dispersible Tablet Platforms Containing Zinc Sulfate and Paracetamol Reconstituted in a Breast Milk Substitute. Pharmaceutics.

[30] Lopalco A., Manni A., Keeley A., Haider S., Li W., Lopedota A., Altomare C.D., Denora N., Tuleu C. (2022). In Vivo Investigation of (2-Hydroxypropyl)-β-cyclodextrin-Based Formulation of Spironolactone in Aqueous Solution for Paediatric Use. Pharmaceutics.

[31] Muoka L.C., Ross S.A., Mithu M.S.H., Nandi U., Douroumis D. (2021). Comparative taste-masking evaluation of microencapsulated bitter drugs using Smartseal 30D and ReadyMix for paediatric dosage forms. AAPS PharmSciTech.

[32] Diezi L., Dao K., Jullien V., Roussel-Maupetit C., Burton I., André P., Bardinet C., Rothuizen L.E., Chtioui H., Manso-Silvan M.A. (2023). An innovative ethosuximide granule formulation designed for pediatric use: Comparative pharmacokinetics, safety, tolerability, and palatability profile versus reference syrup. Pharmacol. Res. Perspect..

[33] Walsh J., Cram A., Woertz K., Breitkreutz J., Winzenburg G., Turner R., Tuleu C., Initiative E.F. (2014). Playing hide and seek with poorly tasting paediatric medicines: Do not forget the excipients. Adv. Drug. Deliv. Rev..

[34] Hu S., Liu X., Zhang S., Quan D. (2023). An Overview of Taste-Masking Technologies: Approaches, Application, and Assessment Methods. AAPS PharmSciTech.

[35] Shah P.P., Mashru R.C. (2008). Formulation and evaluation of primaquine phosphate taste-masked rapidly disintegrating tablet. J. Pharm. Pharmacol..

[36] Molinary S.V., Quinlan M.E. (2012). Sucralose. Sweeteners and Sugar Alternatives in Food Technology.

[37] Mennella J.A., Finkbeiner S., Lipchock S.V., Hwang L.D., Reed D.R. (2014). Preferences for salty and sweet tastes are elevated and related to each other during childhood. PLoS ONE.

[38] Mennella J.A., Bobowski N.K. (2015). The sweetness and bitterness of childhood: Insights from basic research on taste preferences. Physiol. Behav..

[39] Bobowski N., Mennella J.A. (2017). Personal Variation in Preference for Sweetness: Effects of Age and Obesity. Child. Obes..

[40] Petty S., Salame C., Mennella J.A., Pepino M.Y. (2020). Relationship between Sucrose Taste Detection Thresholds and Preferences in Children, Adolescents, and Adults. Nutrients.

[41] Ayenew Z., Puri V., Kumar L., Bansal A.K. (2009). Trends in pharmaceutical taste masking technologies: A patent review. Recent. Pat. Drug. Deliv. Formul..

[42] Banek K., DiLiberto D.D., Webb E.L., Smith S.J., Chandramohan D., Staedke S.G. (2021). Exploring Barriers and Facilitators of Adherence to Artemisinin-Based Combination Therapies for the Treatment of Uncomplicated Malaria in Children in Freetown, Sierra Leone. Healthcare.

[43] Beer N., Ali A.S., Rotllant G., Abass A.K., Omari R.S., Al-mafazy A.W., Björkman A., Källander K. (2009). Adherence to artesunate-amodiaquine combination therapy for uncomplicated malaria in children in Zanzibar, Tanzania. Trop. Med. Int. Health.

[44] Ewing V.L., Terlouw D.J., Kapinda A., Pace C., Richards E., Tolhurst R., Lalloo D.G. (2015). Perceptions and utilization of the anti-malarials artemether-lumefantrine and dihydroartemisinin-piperaquine in young children in the Chikhwawa District of Malawi: A mixed methods study. Malar. J..

